# MiR-125a-5p in MSC-derived small extracellular vesicles alleviates Müller cells injury in diabetic retinopathy by modulating mitophagy via PTP1B pathway

**DOI:** 10.1038/s41420-025-02439-3

**Published:** 2025-05-08

**Authors:** Cong Liu, Jinjin Xiang, Yueqin Chen, Chang He, Jun Tong, Yinglin Liao, Huangyi Lei, Lingyun Sun, Genhong Yao, Zhenggao Xie

**Affiliations:** 1 Department of Ophthalmology, Nanjing Drum Tower Hospital, Affiliated Hospital of Medical School, Nanjing University, Nanjing, 210008 China; 2 Department of Ophthalmology, Jiangdu People’s Hospital Affiliated to Medical College of Yangzhou University, Yangzhou, 225200 China; 3 Department of Ophthalmology, The First Affiliated Hospital of Soochow University, Suzhou, 215000 China; 4 Department of Rheumatology and Immunology, Nanjing Drum Tower Hospital, Affiliated Hospital of Medical School, Nanjing University, Nanjing, 210008 China

**Keywords:** Mesenchymal stem cells, Retinal diseases

## Abstract

Diabetic retinopathy (DR) ranks among the primary causes of adult blindness globally. Oxidative stress and mitochondrial dysfunction play a critical role in the progression of DR. Mounting data indicated that small extracellular vesicles (sEVs) of mesenchymal stem cell (MSC) have the ability to transport bioactive chemicals to target cells, leading to changes in their phenotype. Nevertheless, it remains elusive how MSC-derived sEVs regulate oxidative stress and mitochondrial function in DR. MSC-sEVs was intravitreally injected to streptozotocin (STZ)-treated Sprague-Dawley rats to assess the therapeutic effects on DR. The underlying regulatory mechanism was investigated by coculturing advanced glycation end-products (AGEs)-induced rat Müller cells with/without PTP1B overexpression with MSC-sEVs in vitro, with or without miR-125a-5p suppression. Intravitreal injection of MSC-sEVs improved histological morphology and blood-retinal barrier function, alleviated Müller gliosis, decreased PTP1B expression, redox stress and apoptosis in retina of diabetic rat. MSC-sEVs decreased the accumulation of ROS and improved the structure and function of mitochondria of Müller cells with AGEs treatment. Mechanically, MSC-sEVs activated the mitophagy of AGEs-treated Müller cells, represented by an increased expression of the LC3II/LC3I ratio, TOM20, PINK1 and Parkin along with a decreased expression of P62. Importantly, miR-125a-5p inhibitor abolished the protective effects of MSC-sEVs. Furthermore, the overexpression of PTP1B in Müller cells reduced the effects of MSC-sEVs. These findings suggested that miR-125a-5p of MSC-sEVs alleviates Müller cells injury in DR by modulating PINK1/Parkin-mediated mitophagy via PTP1B pathway.

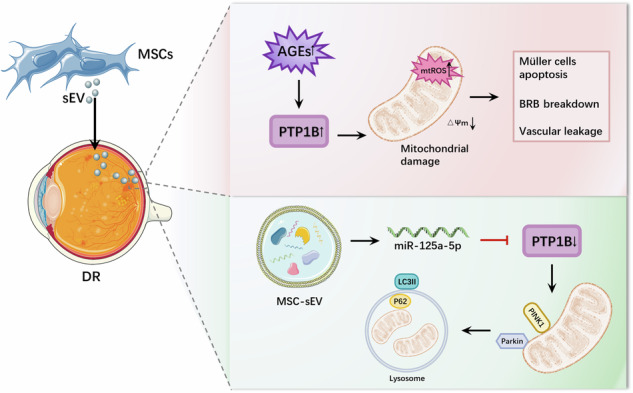

## Introduction

There are currently around 537 million adults globally who have diabetes, which accounts for around 1 in 10 adults. This number is anticipated to rise to 783 million during the following 15 years [1]. Diabetic retinopathy (DR) is the primary ocular condition associated with diabetes mellitus, and has a substantial influence on worldwide healthcare [2]. While DR may initially show no symptoms and progress silently, it can rapidly advance to a stage that poses a threat to eyesight if being untreated. This can ultimately result in permanent vision loss. DR is marked by the gradual deterioration of blood vessels and neurons within the retina, which causes retinal dysfunction, blood-retinal barrier (BRB) breakdown and retinal neovascularization.

Müller cells are essential for maintaining the integrity of the inner BRB and supporting the function of retinal neurons [3]. Müller cells provide a physical connection between retinal neurons and areas that need to exchange molecules, such as retinal blood vessels, vitreous humor, and the subretinal space. Dysfunction of Müller cells can trigger the occurrence of edema and neurodegeneration, which potentially occur earlier than microvascular remodeling [4]. Increased glucose levels induce oxidative stress and glial abnormal activation in Müller cells [5].

Oxidative stress is a significant contributor to the progression of DR. Excessive generation of reactive oxygen species (ROS) can damage the tissue surrounding retinal capillaries, ultimately resulting in DR [6]. Specifically, epigenetic alterations can lead to oxidative stress that persists for an extended period, even when blood glucose levels return to normal. This phenomenon is referred to as “metabolic memory” [7]. Prolonged exposure to consistently elevated levels of glucose in the blood leads to mitochondrial damage, while increasing generation of free radicals in the mitochondria contributes to significant metabolic abnormalities related to the onset of DR. The main characteristics of mitochondrial dysfunction include decreased adenosine triphosphate (ATP) synthesis and mitochondrial membrane potential (MMP), overproduction of ROS, disruptions in mitochondrial autophagy, and aberrant mitochondrial dynamics[8]. Mitophagy is a vital mechanism that selectively detects and eliminates damaged or superfluous mitochondria through autophagy, hence playing a crucial role in maintaining mitochondrial equilibrium [9].

Currently, there has been a surge of interest in mesenchymal stem cells (MSCs) as an effective approach for various eye disorders by improving angiogenesis, decreasing oxidative stress damage and accelerating tissue repair [10, 11]. Accumulating studies have demonstrated that the majority of MSC effects are paracrine in nature and are facilitated by MSC derived small extracellular vesicles(sEVs) [12, 13]. MSC-derived sEVs have demonstrated significant potential in enhancing mitochondrial function [14]. However, the therapeutic benefits and mechanisms of MSC-sEVs on mitophagy remain to be elucidated, as far as we know, especially concerning DR. This study explored the therapeutic effects and mechanisms of sEVs released by MSCs on both STZ-induced diabetic rat model and AGEs-induced Müller cells.

## Results

### MSC-sEVs reduced retinal histological damage and regulated retinal vascular dysfunction in STZ-induced diabetic rats

**Fig. 1:**
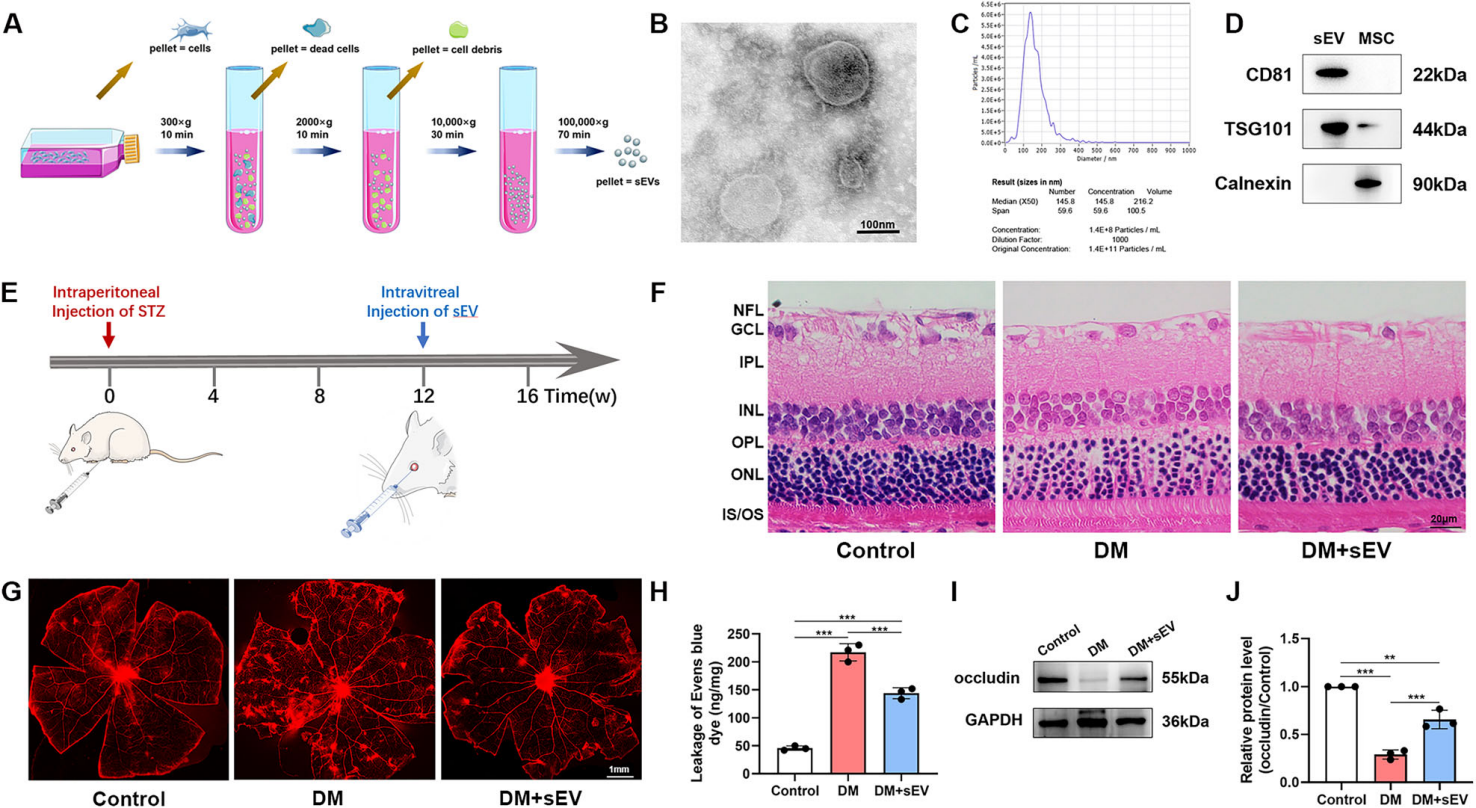
Intravitreal injection of MSC-sEVs regulated retinal vascular dysfunction in STZ-induced diabetic rat. **A** Flow chart for the sEV purification procedure based on differential ultracentrifugation. **B** Morphology of sEV under TEM (scale bar=100 nm). **C** Determination of sEV particle size by NTA analysis. **D** Western blot analysis of sEV surface marker proteins. **E** Schematic diagram of animal treatment. **F** Representative retinal H&E staining images for the 3 groups (scale bar = 20 μm). **G** Representative images of EB staining showing retinal vascular leakage in rats (scale bar = 1 mm). **H** The quantity of the Evans Blue leakage was determined (*n* = 3). **I, J** Protein expression levels of occludin in the retina, as determined by western blot (*n* = 3). *P < 0.05, **P < 0.01, ***P < 0.001.

MSC-sEVs were acquired from hUC-MSC conditioned medium using a two-step ultracentrifugation technique (Fig. 1A). The obtained samples were then analyzed using transmission electron microscopy (TEM), nanoparticle tracking analysis (NTA), and western blot test to determine their characteristics. The TEM pictures showed that the particles were spherical and surrounded by a double-layered membrane. The sizes of the particles ranged from around 100 to 200 nm, as shown in Fig. 1B. The results revealed by the NTA test are as follows: a peak size of 145.8 nm and a concentration of 1.4 × 10^11^ particles/mL (Fig. 1C). The results of western blot demonstrated the existence of characteristic surface markers of sEVs, including CD81, TSG101 and Calnexin, in MSC-sEVs (Fig. 1D).

Rats received intraperitoneal injections of STZ to establish a diabetic model. Figure1E illustrates the establishment of the DR rat model via intraperitoneal injection of STZ at a dosage of 60 mg/kg. After 12 weeks following STZ injection, MSC-sEVs were administered into the vitreous cavity of diabetic rats. The protective effect of MSC-sEVs on hyperglycemia-induced diabetic retinal structures was investigated. H&E staining revealed the cells within the inner nuclear layer (INL) and outer nuclear layer (ONL) exhibited a normal arrangement and closely packed manner in the control group. Whereas, structure of retina was significantly abnormal in the DM group, particularly evident in the swelling of the nerve fiber layer (NFL) and disorganized arrangement of cells in the ganglion-cell layer (GCL), INL and ONL. These structure abnormalities were ameliorated in DM with MSC-sEVs treatment group (Fig. 1F). In addition to histologic evidence, retinal vascular leakage was used to assess the efficacy of the different treatments. EB staining demonstrated that vascular leakage was greatly decreased by MSC-sEVs treatment (Fig. 1G, H). To assess the effects of MSC-sEVs on the tight junction protein within the retina, western blot was conducted to evaluate the expression of occludin. The expression of occludin protein was notably reduced in the DM group in comparison to the control group. In the DM+sEV group, the expression of the occludin protein was considerably elevated compared with the DM group (Fig. 1I, J).

### MSC-sEVs alleviated oxidative stress damage and apoptosis in the retina of diabetic rats

**Fig. 2:**
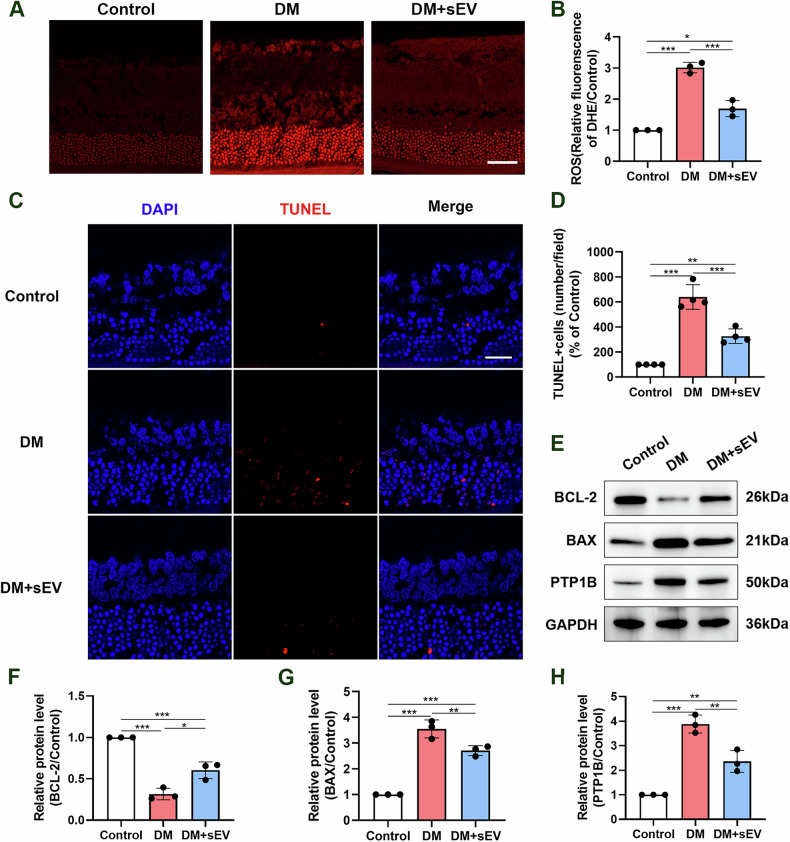
Intravitreal injection of MSC-sEVs alleviated oxidative stress damage and apoptosis in the retina of diabetic rats. **A** Immunofluorescence staining of rat retina using DHE. Red fluorescence represented ROS production (scale bar = 30 μm). **B** Measurement of the intensity of red fluorescence (*n* = 3). **C** Representative images of TUNEL (red) and DAPI (blue) in retinal sections in each group (scale bar = 15 μm). **D** The bar graph depicts the mean percentages of apoptotic cells (*n* = 4). **E-H** Protein expression levels of BCL-2, BAX and PTP1B in retina, as determined by western blot (*n* = 3). *P < 0.05, **P < 0.01, ***P < 0.001.

Superoxide facilitates the conversion of DHE into ethidium bromide. Ethidium bromide is capable of intercalating into DNA and has been employed as an indirect means of production [15]. The rat retina of the DM group displayed a significant upregulation of fluorescence intensity relative to the normal group. Additionally, the sections from the DM+sEV group showed a decrease of DHE reaction, indicating superior treatment efficacy (Fig. 2A, B). Nevertheless, the precise mechanism of hyperglycemia-induced damage in retina remains undefined. Elevated level of ROS is characteristic of the initial phases of apoptosis. In this study, the degree of retinal cell apoptosis was assessed using TUNEL labelling of the retinal sections. According to TUNEL results, the number of TUNEL-positive retinal cell counts of DM group was substantially higher than that of the control group, whereas the number of apoptotic cells of DM+sEV group showed a tendency of decline (Fig. 2C, D). Subsequently, we conducted an analysis of apoptosis-related proteins, specifically focusing on the anti-apoptotic protein BCL-2 and the pro-apoptotic protein BAX. Figure 2E-G revealed that MSC-sEVs increased anti-apoptotic proteins while decreased apoptosis-related proteins in retinas of DM rats.

Protein tyrosine phosphatases (PTPs) are crucial for insulin signaling pathways [16]. PTP1B phosphatase activity has been found to be higher in numerous diabetes complications, such as DR [17]. To validate the differential expression of PTP1B among different groups, PTP1B protein levels were assessed by western blot. The results showed a notable downregulation of PTP1B in DM+sEV group relative to the DM group (Fig. 2H).

### MSC-sEVs alleviated the reactive gliosis of Müller cells in the retinas of diabetic rats

**Fig. 3:**
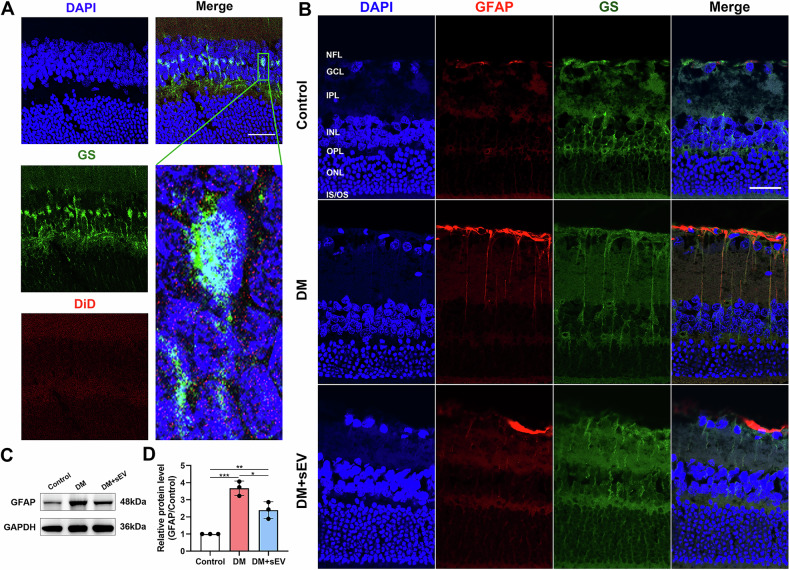
Intravitreal injection of MSC-sEVs alleviated the reactive gliosis of Müller cells in the retinas of diabetic rats. **A** In vivo sEVs uptake analysis. DiD labeled MSC-sEVs were locally injected into the vitreous body. Representative immunostaining of DiD (red), GS (green) and DAPI (blue) in retinal sections (scale bar = 30 μm). **B** Representative immunostaining of GFAP (red) and DAPI (blue) in retinal sections in each group. NFL nerve fiber layer, GCL ganglion cell layer, IPL inner plexiform layer, INL inner nuclear layer, OPL outer plexiform layer, ONL outer nuclear layer, IS/OS inner segment/outer segment, GS glutamine synthetase (scale bar = 20 μm). **C**, **D** Protein expression levels of GFAP in retina, as determined by western blot (*n* = 3). *P < 0.05, ***P < 0.001.

MSC-sEVs were stained with the red fluorescent cell linker DiD, a widely used dye for sEVs, and were then delivered into the retina through intravitreal injection. Significantly, DiD positive sEVs were detectable in Müller cells after the injection, as evidenced by the colocalization of DiD labeled sEVs with GS-positive Müller cells. This result confirmed that MSC-sEVs can be internalized by Müller cells in vivo (Fig. 3A).

GFAP is mainly expressed by retinal Müller cells, and its activation responds to changes of the retinal pathological condition. Figure 3B demonstrated that GFAP protein (shown by the red signal) appeared primarily on the vitreous side of the healthy retina, with minimal occurrence of filamentous protein production on the surface. In the DM group, there was an upregulation of GFAP expression. Additionally, a whisker-like structure was observed, oriented perpendicular to the vitreous surface of the retina. Compared with DM group, the numbers of red GFAP filaments were decreased in the nerve fiber and ganglion cell layers in the DM+sEV group. In comparison to the DM group, the DM+sEV group exhibited a reduction in the quantities of GFAP filaments in both the nerve fiber layer and ganglion cell layer. Moreover, western blots were conducted using anti-GFAP antibody on retinas. The results indicated that GFAP levels were reduced in the DM+sEV group in comparison to the DM group (Fig. 3C, D). These findings suggested that MSC-sEVs improved retinal gliosis following DM.

### SEVs inhibited AGEs-induced oxidative stress and apoptosis through PTP1B pathway

**Fig. 4:**
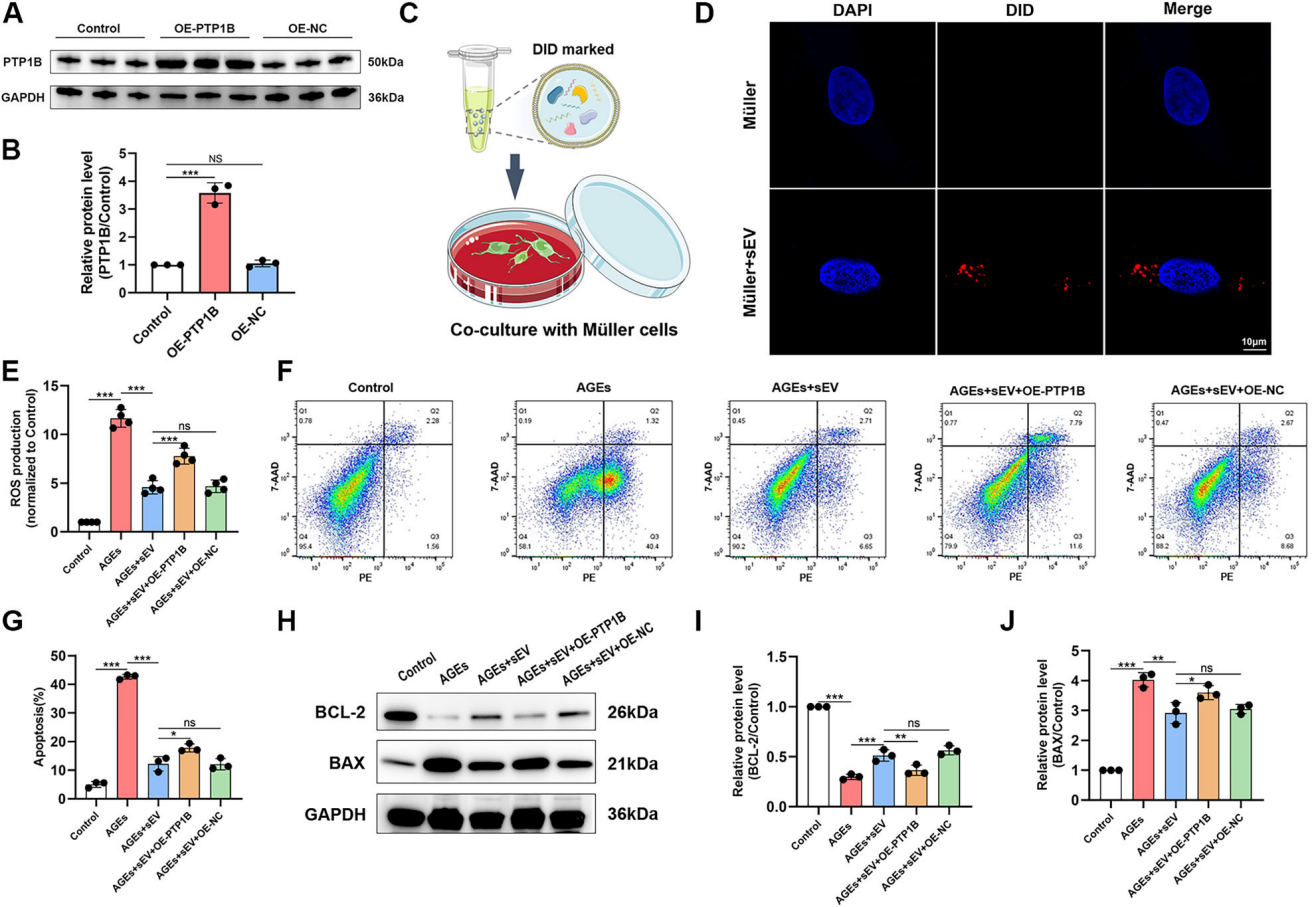
sEVs inhibited AGEs-induced oxidative stress and apoptosis through PTP1B pathway. **A, B** Protein expression levels of PTP1B in Müller cells, as determined by western blot (*n* = 3). **C** Schematic diagram of cell treatment. **D** Immunofluorescence staining of the uptake of DiD-labeled sEVs (red) in Müller cells (scale bar = 10 μm). **E** The intracellular levels of ROS were measured (*n* = 4). **F**, **G** Flow cytometry analysis of pericytes for apoptosis and quantification (*n* = 3). **H-J** Protein expression levels of BCL-2 and BAX in Müller cells, as determined by western blot (*n* = 3). *P < 0.05, **P < 0.01, ***P < 0.001.

To elucidate the potential mechanisms by which PTP1B contributes to oxidative stress and apoptosis, we choose Rat Müller cells for following lentivirus transfection. The western blot demonstrated a clear overexpression of the PTP1B protein (Fig. 4A, B). To evaluate the endocytic effects of sEVs on Müller cells, we labeled the sEVs with DiD and then co-incubated them with Müller cells for 24 h (Fig. 4C). Through the utilization of high-magnification fluorescence microscopy, we observed sEVs, demonstrating the process of endocytosis (Fig. 4D).

Hyperglycemic circumstances lead to the accumulation of advanced glycation end products (AGEs), which are believed to have a significant impact on the development of DR [6]. Müller cells, with or without PTP1B overexpression, were exposed to 300 μg/ml of AGEs to simulate the diabetic microenvironment. Subsequently, they were co-cultured with MSC-sEVs for 48 h and the ROS levels of cells were measured. As shown in Fig. 4E, ROS generation was dramatically increased following treatment with AGEs and this tendency was reversed by sEVs.

Since ROS overexpression is a crucial factor in the process of mitochondrial apoptosis [18], we employed Annexin V-PE/7-AAD double staining to validate the presence of apoptosis in Müller cells. Figure 4F, G demonstrates that the number of apoptotic cells, including early apoptotic (Annexin V-PE^+^/7-AAD^-^) and late apoptotic (Annexin V-PE^+^/7-AAD^+^) cells increased in Müller cells following treatment with AGEs. In line with the aforementioned findings, the apoptosis-related proteins in AGEs-exposed Müller cells were similarly modified (Fig. 4H-J), with a reduction of anti-apoptotic protein BCL-2 and elevations of pro-apoptotic proteins BAX. Subsequently, sEVs successfully reversed this effect. Noteworthy, PTP1B overexpression partially cabrogated the antioxidant and anti-apoptosis effect of sEVs in Müller cells. These results collectively demonstrated that sEVs had a protective impact in reducing oxidative stress and apoptosis by blocking the PTP1B signal pathway in vitro.

### MSC-sEVs regulated PTP1B pathway in Müller cells via delivery of miR-125a-5p

**Fig. 5:**
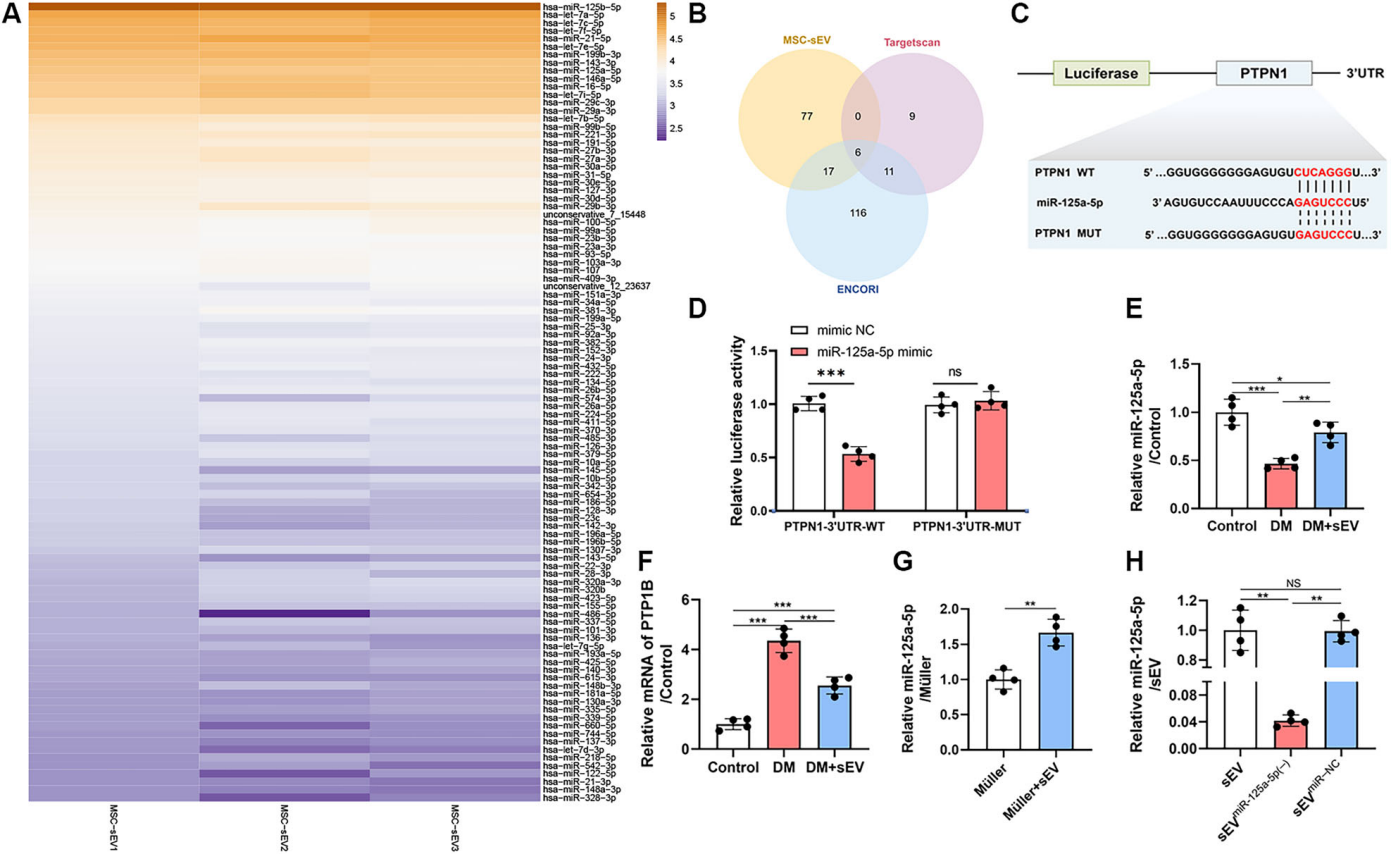
MSC-sEV-miR-125a-5p was the candidate effector small RNA sequencing for analysis of miRNAs in MSC-sEVs. **A** Heatmap shows top 100 miRNAs in MSC-sEVs. **B** Venn diagrams illustrate the overlapping regions of three sets (Top 100 miRNAs of MSC-sEVs, miRNAs predicted by TargetScan database and ENCORI database) **C**, **D** Luciferase reporter assay for validating the interaction of miR-125a-5p with the 3′ UTR of PTPN1 (*n* = 3). **E**, **F** Determination of the expression levels of miR-125a-5p and PTP1B in retina by qRT‐PCR (*n* = 4). **G** Relative expression of miR-125a-5p mRNA in Müller cells cocultured with sEVs by qRT-PCR (*n* = 4). **H** Relative miR-125a-5p level in sEVs with miR-125a-5p inhibition (sEV^miR-125a-5p(−)^) and negative control (sEV^miR−NC^) by qRT-PCR (*n* = 4).

In the preceding research, MSC-sEVs had the ability to interfere with the expression of PTP1B in retina. However, further investigation is needed to identify the specific molecules within MSC-sEVs that are responsible for this regulatory effect. MiRNA, along with other components, is widely recognized as one of the most crucial cargos that sEVs deliver to destination cells [19]. In order to determine the specific miRNAs that provided the protective effects of MSC-sEVs on DR, we examined total small RNA sequencing to analyze the RNA profiles of MSC-sEVs (Supplementary 1) (Fig. 5A). Subsequently, we employed the TargetScan database to forecast the miRNAs responsible for the suppression of PTP1B, resulting in the identification of 26 miRNAs. In addition, we utilized the ENCORI database to forecast the targets, resulting in the identification of 150 miRNAs. In order to identify the specific candidate miRNA, we conducted an analysis of the three databases mentioned above and found a total of 6 miRNAs were present in all of them (Fig. 5B). Then, the identified target miRNAs underwent additional examination. In addition, the online freeware BiBiServe RNAhybrid was utilized to compute the minimal free energy (MFE) of the miRNAs when they were matched with the 3′UTR of PTP1B mRNA (Supplementary 2. Table S1) [20]. MiR-125a-5p was emerged as the leading contender due to its lower free energy. In order to investigate the specific interaction between miR-125a-5p and PTP1B, luciferase assay was utilized (Fig. 5C). The results demonstrated that miR-125a-5p specifically suppressed the expression of wild‐type PTPN1 3′UTR, while having no effect on the mutant PTPN1 3′‐UTR (Fig. 5D). Moreover, the DM+sEV group exhibited elevation of miR-125a-5p and reduction of PTP1B mRNA in the retinal tissues, relative to the DM group (Fig. 5E, F). Meanwhile, sEVs were cocultured with Müller cells for 48 h to investigate their potential as carriers of miR-125a-5p. The results indicated that Müller cells cocultured with sEVs exhibited elevated level of miR-125a-5p compared to Müller cells cultured alone (Fig. 5G). These findings suggested the significant role of MSC-sEV-miR-125a-5p in suppressing PTP1B.

### MSC-sEV-derived miR-125a-5p mitigated mitophagy and improved mitochondrial function in AGEs-induced Müller cells

**Fig. 6:**
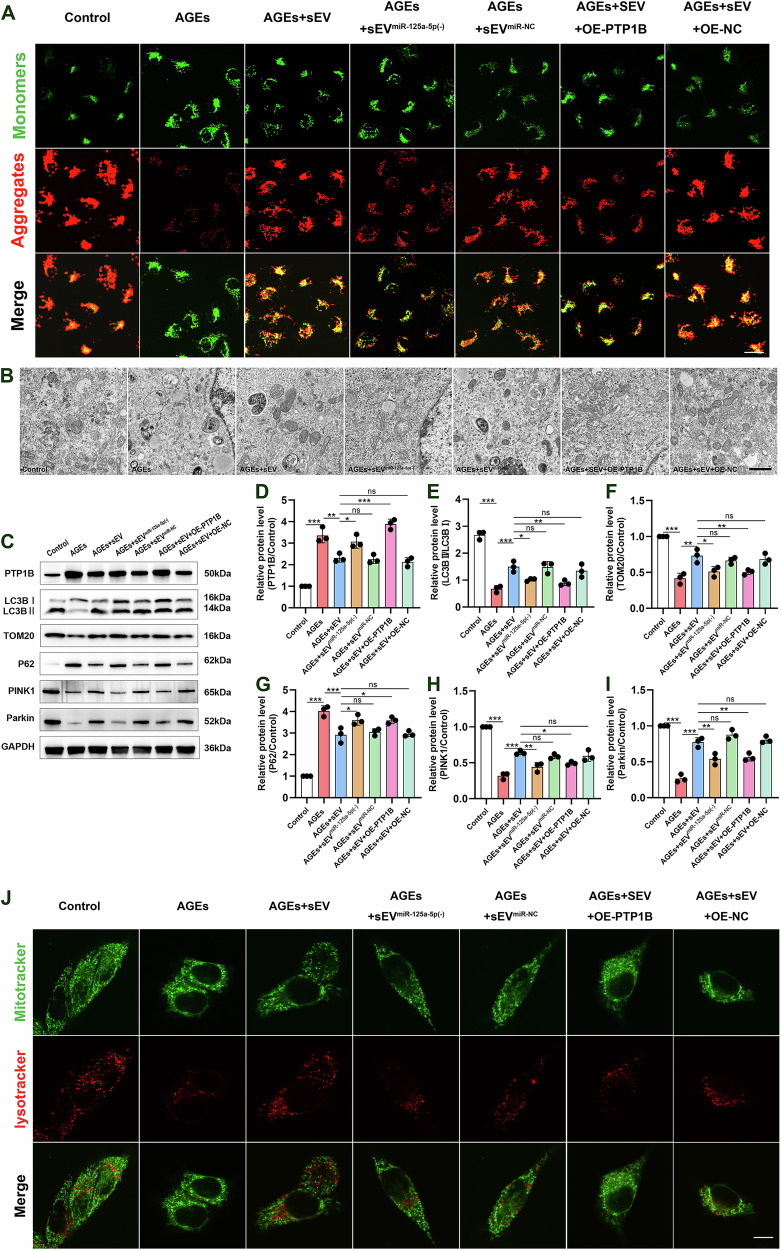
MSC-sEV-derived miR-125a-5p promoted the amelioration of mitophagy and improved mitochondrial function in AGEs-induced Müller cells. **A** Representative image using the cationic dye JC-1, displaying red fluorescent J-aggregates and green J-monomers (scale bar = 30 μm). **B** Transmission electron microscopy images of mitochondria and autophagic vesicles in rat muller cells. Typical TEM images of cell ultrastructure (scale bar = 1 μm). **C-I** Protein expression levels of PTP1B, LC3II/LC3I, TOM20, P62, PINK1 and Parkin in Müller cells, as determined by western blot (*n* = 3). *P < 0.05, **P < 0.01, ***P < 0.001. **J** Mitochondria and lysosomes stained by MitoTracker-Red and LysoTracker-Green, respectively (scale bar = 10 μm).

Increased PTP1B activity has been shown to interfere with mitochondrial function and glucose metabolism [10]. We next explored the mechanism by which MSC-sEVs transport miR-125a-5p to inhibit PTP1B and improve oxidative stress in DR. Mitochondrial membrane potential (MMP) is an indicator of mitochondrial activity, and a decrease in MMP is strongly linked to an increase in mitochondrial ROS generation. The detection of MMP was determined by JC-1 labeling, which produces red fluorescence when healthy mitochondria is present while green fluorescence when there is mitochondrial dysfunction. To assess the role of miR-125a-5p in MSC-sEVs, MSCs were infected with LV-miR-125a-5p inhibitor to block miR-125a-5p in MSC-sEVs. Subsequently, sEV^miR-125a-5p (-)^ was isolated. The qRT-PCR assay demonstrated that sEV^miR-125a-5p(-)^ displayed a markedly decreased level of miR-125a-5p in comparison to both sEV and sEV^miR-NC^ and no substantial difference was observed between sEV and sEV^miR-NC^ (Fig. 5H). The Müller cells induced by AGEs were treated with sEV, sEV^miR-125a-5p(-)^, sEV^miR-NC^ for 48 h. The presence of AGEs resulted in an augmentation of green fluorescence and a reduction in red fluorescence, indicating a decline in MMP levels. The following use of sEVs treatment could mitigate this loss. Meanwhile, the treatment with sEV^miR-125a-5p(-)^ or when Müller cells overexpressed PTP1B resulted in a significantly decrease of MMP in comparison to either sEV or sEV^miR-NC^. Nevertheless, there was no notable distinction between sEV and sEV^miR-NC^ (Fig. 6A).

In order to validate the aforementioned discoveries at the ultra-microstructural level, we examined the alterations in mitochondria using transmission electron microscopy (TEM). TEM examination of Müller cells revealed mitochondrial defects including fragmentation, swelling, loss of mitochondrial cristae and a reduction in the number of autophagosome vacuoles containing mitochondria upon AGEs exposure. The sEVs therapy offered a shield for mitochondria from AGEs damage. Furthermore, the therapeutic efficacy will be partially reduced by the presence of sEV^miR-125a-5p(-)^ or by the increased expression of PTP1B in Müller cells (Fig. 6B).

Accurate control of mitophagy is essential for preserving the optimal number of functional mitochondria within cells [21]. We evaluated the proteins related to mitochondrial function and mitophagy in Müller cells. After treating Müller cells with AGEs, we observed a significant downregulation of the levels of TOM20, LC3II/LC3I, PINK1, and Parkin proteins. Conversely, the levels of P62 were elevated. Treatment of sEVs can effectively reverse the protein expression. However, the alterations of these proteins were partly counteracted by sEV^miR-125a-5p(-)^ or overexpression of PTP1B in Müller cells (Fig. 6C-I). Furthermore, the co-localization of mitochondria and lysosome was reduced after exposure to AGEs, while treatment of sEVs obviously enhanced overlapping signals of MitoTracker and LysoTracker, indicating that sEVs regulate mitophagy of Müller cells. Noteworthy, the changes in the trend were partly mitigated by sEV^miR-125a-5p(-)^ or by the overexpression of PTP1B in Müller cells (Fig. 6J). Collectively, these results suggested that by releasing miR-125a-5p to Müller cells, sEVs repressed PTP1B expression and enabled improvement of mitophagy that alleviated mitochondrial injury in response to AGEs.

## Discussion

The objective of this study was to examine the protective effect of intravitreal injection of MSC-sEVs on STZ-induced diabetic rat. Our findings demonstrated that MSC-sEVs mitigated oxidative stress and apoptosis by inhibiting PTP1B pathway and reversed retinal damage resulting from prolonged hyperglycemia. This was also verified through in vitro tests. Furthermore, our findings indicated that miR-125a-5p in MSC-sEVs partially contributed to the beneficial effects of MSC-sEVs on DR by modulating PINK1/Parkin-mediated mitophagy via PTP1B pathway.

Müller cells, which span the entire retinal tissue, interact with cells in all retinal layers and play important roles in formation, maintenance, and enhancement of the BRB. Müller dysfunction may contribute to a breakdown of the BRB and vascular alterations, two distinguishing features of DR pathology. Our study observed a decrease in the expression of tight junction protein occludin and aggravation of vascular leakage in the retinas of DR rats, consistent with previously published findings [22]. This is corroborated by studies that showed that reactive gliosis can happen in response to hyperglycemia characterized by an upregulated level of GFAP, a marker of glial cell activation [23]. Our results of western blot and immunofluorescence assay of GFAP in rat retinal tissues confirmed the previous findings. Research has proven that long-term hyperglycemia exposure to elevated glucose levels can damage Müller cells, leading to inflammation, oxidative stress and apoptosis. In the current study, the co-localization of GS-labeled Müller cells with DiD-labeled MSC-sEVs demonstrated that retinal Müller cells absorbed sEVs. Additionally, administering MSC-sEVs into the vitreous cavity of diabetic rats resulted in a reduction of GFAP expression, increased occludin expression, as well as relief of vascular leakage.

Dysregulation in redox homeostasis contributes to the onset and progression of DR [24]. Our results revealed that there were notably increased levels of ROS in either Müller cells stimulated by AGEs or the retinas of diabetic rats. Oxidative stress, resulting from metabolic instability in a high glucose environment, is the primary mechanism responsible for mitochondrial damage and can potentially trigger mitochondrial pathway apoptosis [25]. Within the present study, we distinctly observed the occurrence of apoptosis in Müller cells stimulated by AGEs and in retina tissues of STZ-induced diabetic rats, as evidenced by flow cytometry, and western blot. Our results also confirmed the above findings with the discovery that treatment with AGEs led to an elevation in BAX expression and a decrease in the level of BCL-2. Thus, the most usually employed treatments involve scavenging ROS, blocking their production, or enhancing the antioxidant capacity of cells. While the ability of MSC-sEVs to reduce oxidative stress has been documented in multiple studies, its specific impact on DR remains inadequately explored [26]. Our findings demonstrated that MSC-sEVs treatment effectively downregulated ROS levels and reduced the apoptotic rate both in vivo and in vitro. ROS are the primary free radicals in oxidative systems, and excessive generation of ROS can result in impairment of DNA, RNA, and protein. Around 90% of cellular ROS originate from mitochondria [27]. The role of mitochondrial function is crucial in maintaining the metabolic processes and balance in the retina, which is recognized as one of the tissues with the highest metabolic demands, surpassing the metabolic rate of brain tissue [28]. Multiple researches have indicated that the mitochondria dysfunction could be induced by DR along with the decrease of mitochondrial membrane potential as well as the accumulation of ROS [27, 29]. Repairing mitochondrial damage is a key strategy for treating DR. Our current study observed a decrease of mitochondrial membrane potential in Müller cells induced by AGEs. Simultaneously, the ultrastructure revealed vacuolated mitochondria, accompanied by disruption of the lamellar cristae.

Mitophagy, which selectively eliminates damaged mitochondria from the cell using the autophagy pathway, is intimately associated with mitochondrial quality control and homeostasis [30]. PINK1 is a well-established protein that is vital for regulating the mitophagy signaling pathway. When mitochondria undergo damage, PINK1 gathers in the outer mitochondrial membrane and triggers the activation and recruiting of Parkin [31]. Our findings revealed that the treatment of AGEs resulted in a decrease in the expressions of PINK1 and Parkin, as well as downregulation of the LC3II/LC3I ratio, while simultaneously causing the accumulation of p62, which serves as a substrate for mitophagy-mediated degradation in Müller cells. Of note, intervention of MSC-sEVs successfully reversed the aforementioned tendency and improved the structure and function of mitochondria.

PTP1B is recognized for its adverse regulating influence on leptin and insulin signaling, leading to substantial implications for the development of metabolic diseases [17]. Researchers have discovered links between the activity of PTP1B phosphatase and DR [32]. Previous study showed that patients with proliferative diabetic retinopathy (PDR) exhibited elevated levels of PTP1B in the vitreous humor [33]. Our findings suggest that diabetic rats’ retinas and Müller cells induced by AGEs had higher PTP1B levels than controls. PTP1B plays a vital part in regulating oxidative stress in various tissues through both processes of generating and scavenging ROS. Increased PTP1B activity is recognized to interfere with mitochondrial respiration and glucose metabolism [34]. Moreover, there also existed a study that indicated the underlying correlations between PTP1B knockdown and enhanced mitophagic levels in bone marrow mesenchymal stem cells [35]. In the present study, we subjected Müller cells to lentiviral transfection in order to promote overexpression of PTP1B and found the effect of MSC-sEVs was weakened. Our studies suggested that MSC-sEVs suppressed PTP1B expression, leading to the activation of PINK1/Parkin pathway and effectively attenuated AGEs-induced mitochondrial dysfunction.

MiRNAs are a well-established category of noncoding single-stranded RNAs that regulate RNA targets via post-transcriptional mechanisms [36]. Accumulating evidence demonstrates that miRNAs, a significant component of sEV, play a pivotal role in facilitating stem cell functionality via being delivered to recipient cells [37]. In this study, to find out which kind of miRNA regulate PTP1B pathway, we conducted miRNA sequencing of MSC-sEVs and utilized online bioinformatic software to identify miRNAs that target PTP1B. Notably, MSC-sEVs enrich miR-125a-5p, a putative target of PTP1B. Following that, dual luciferase reporter analysis was used to confirm the regulatory impact of miR-125a-5p on PTP1B. Subsequent qRT-PCR analysis verified that diabetic rats had reduced levels of miR-125a-5p in the retina in comparison to the control rats. The administration of MSC-sEVs was capable of reversing this tendency. To get a more profound understanding of the essential role of sEV-miR-125a-5p in the process of repairing mitochondrial damage, MSCs were infected with LV-miR-125a-5p. Through investigation, we found that MSC-sEVs alleviated Müller cells injury in DR by modulating PINK1/Parkin-mediated mitophagy via specific delivery of miR-125a-5p and regulation of PTP1B.

Consistent with the findings of other articles, this study found that sEVs can alleviate hyperglycemia-induced retinal injury. However, our study focused on the different aspects and molecular mechanisms. For instance, Reddy et al. [38] highlighted the potential of MSC-sEVs loaded with anti-vascular endothelial growth factor drug bevacizumab to reduce the frequency of intravitreal injection required for treating DR in a rat model. Chen et al. [39] showed that MSC-sEVs alleviate DR by delivering miR-22-3p to inhibit the NLRP3 inflammasome in microglia. Sun et al. [40] demonstrated that MSC-sEVs loaded with miR-5068 and miR-10228 target the HIF-1α/EZH2/PGC-1α pathway to enhance retinal function in db/db mice. This study presented a novel perspective by focusing on the role of miR-125a-5p in MSC-sEVs and its impact on mitophagy via PTP1B pathway in retinal Müller cells. Our research has certain limitations and unresolved questions. To begin with, only one single dose of MSC-sEVs was injected into the vitreous cavity in this study and additional research is required to elucidate the effects of earlier and repeated administration of sEVs. In addition, in vitro AGEs model does not completely mimic the intricacies of the DR microenvironment. Moreover, further study is necessary to clarify the exact mechanism by which PTP1B modulates the PINK1-Parkin pathway to impede mitophagy, thus delving into the specific processes at play.

In conclusion, this study demonstrated that MSC-sEVs effectively mitigated the severity of DR by inhibiting PTP1B through delivering miR-125a-5p to modulated PINK1/Parkin-mediated mitophagy. Our study suggests that MSC-sEVs could serve as an innovative approach for treating DR.

## Materials and methods

### Culture and transfection of cells

Human umbilical cord MSCs (hUC-MSCs) were from the Stem Cell Center of Jiangsu Province. The detailed purification and identification procedures were described previously [41]. The hUC-MSCs were cultured in DMEM/F12 medium (KGL1201-500; KeyGEN) supplemented with 10% fetal bovine serum (BC-SE-FBS08; BioChannel) and transfected with lentivirus - encoding miR-125a-5p inhibition (TCACAGGTTAAAGGGTCTCAGGGA). GV492 plasmids containing the PTPN1 gene sequence (Accession number: NM_012637) were packaged with viral packaging helper plasmids to construct the lentivirus vector overexpressing PTP1B (LV-OE-PTP1B), where blank GV492 plasmids were set as the negative control (LV-OE-NC) vector. Following this, the Rat Müller cells (CP-R117; Procell) were subjected to infection with either LV-OE-PTP1B or LV-OE-NC according to the producer’s guidelines.

### MSC-sEVs isolation and identification

SEVs were isolated from supernatants of hUC-MSC as previously mentioned [42]. Once cells reached 80% confluence, the medium was substituted with exosome-free medium which was collected after 48 h. The supernatants were subjected to centrifugation at 300 g for 10 min, 2000 g for 10 min, 10,000 g for 30 min and 100,000 × g for 70 min. This process effectively eliminated cells, cell debris, and organelles. The pellet was suspended in PBS and subjected to repeated ultracentrifugation procedures. Three types of MSC-sEVs were obtained: sEV, sEV^miR-125a-5p(-)^, and sEV^miR-NC^ and then preserved at −80 °C before utilization. The concentration and size distribution of sEV was determined utilizing a nanoparticle tracking and NanoSight analysis system.

### Labeling and internalization of MSC-sEVs

To label the sEV solution, it was incubated with DiD (C1039; Beyotime; 5 µg/ml) for 30 min. The samples were rinsed in PBS using ultracentrifugation. The concentrated solutions were then diluted in PBS [43]. The MSC-sEVs tagged with DiD were incubated with cells and subsequently examined using confocal microscopy after 24 h.

### Streptozotocin (STZ)-induced diabetic rats and intravitreal administration of small extracellular vesicles

45 male Sprague-Dawley rats, aged 8 weeks and weighing between 200 and 220 g, were acquired from Yinuojia Biotechnology Corporation (Bengbu, China). The rats were kept in conventional circumstances, with a temperature of 24 ± 2 °C, humidity of 45 ± 5%, and a 12 h light/dark cycle. The Ethical Committee of Nanjing Drum Tower Hospital permitted the protocols. We randomly assigned the rats into the diabetes (*n* = 30) and normal groups (*n* = 15). The rats of diabetic group received an intraperitoneal injection of STZ (S110910; Aladdin; 60 mg/kg in 0.1 M citrate buffered) along with a high-fat diet. The control rats were administered the corresponding amount of citrate buffer with normal diet. SD rats that developed STZ-induced diabetes with blood glucose levels exceeding 16.7 mmol/L, were randomly allocated into 2 groups according to intravitreal injection PBS (*n* = 15) or 1.5 × 10^9^ sEV (*n* = 15). The rats were anesthetized effectively by intraperitoneal injection of sodium pentobarbital before the intravitreal injection. Upon finishing the experiment, all the rats were anesthetized, after which the cervical vertebrae were dislocated resulting in their death.

### H&E staining

The rat eyeballs were removed and immersed in a solution of 4% paraformaldehyde (PFA) and then cut into thin sections (5 μm thick). Subsequently, these sections underwent hematoxylin and eosin staining.

### Assessment of retinal vascular leakage

The rats were administered a deep anesthesia and injected with Evans Blue dye (E2129; Sigma-Aldrich; 30 mg/mL in saline) through the tail vein with a dosage of 45 mg/kg. The eyeballs were removed and preserved in 4% PFA solution for 3 h. The entire retina was removed, delicately placed on a slide, and examined using a fluorescence microscope. Blood–Retinal Barrier breakdown was calculated in all the groups as described previously [44].

### Mitochondrial membrane potential

The JC-1 Mitochondrial Membrane Potential Assay Kit (40706; Yeasen) was utilized to detect the mitochondrial membrane potential. Following the protocol, a functional solution of JC-1 was introduced to the cells and incubated for 30 min at 37 °C in the absence of light. Following 3 washes with PBS, the finished medium was added. The results were visualized using a fluorescence microscope.

### Measurement of autophagosomes and mitochondrial morphology

The medium was removed from the cells, and a 2.5% glutaraldehyde fixative at normal temperature was applied, fully submerging the cells. Next, the cells were immobilized at ambient temperature in the absence of light for 10 min, after which they were delicately scraped in a single direction using a cell scraper at a 45° angle. Transfer the complete cell suspension into the 1.5 ml tube. The rotational speed is set at 200 g for a centrifugal process lasting 5 min. Following the removal of the initial fixative and subsequent addition of a fresh fixative, the cell pellet was delicately gathered and reconstituted in the fixative solution. After postfixes in 1% osmium tetroxide, cells were dried in acetone and dyed with 2% uranyl acetate. The blocks were sliced and shaped using an ultramicrotome to achieve a thickness of 70 nm. Afterwards, toluidine blue staining was conducted, and images were obtained using a Hitachi transmission electron microscope.

### Evaluation of lysosome and mitochondria colocalization

Mitochondria and lysosome of live cells were labeled with Mito-Tracker Red (C1032; Beyotime) and Lyso-Tracker Green (C1047S; Beyotime) 37 °C for 20 min.

### Immunofluorescence

Frozen sections of eyes were blocked with 10% goat serum for 1 h, and incubated overnight at 4 °C with primary antibodies anti-GFAP (A19058; 1:100; Abclonal) or anti-GS (A5437; 1:100; Abclonal), respectively. Following a 1 h incubation at room temperature, secondary antibodies were applied, followed by DAPI staining.

### ROS content detection

Dihydroethidium (DHE) is a widely used fluorescent probe for the measurement of superoxide anion levels. Therefore, in our study, the sections of eyes were stained with DHE (S0064S; Beyotime). Then, the sections were sealed with anti-fluorescence quencher after 30 min at 37 °C. Confocal microscopes (594 nm) captured fluorescent pictures. The fluorescence intensity of DHE red was quantified using ImageJ. ROS Assay Kit (S0035S; Beyotime) was used to measure the intracellular ROS levels in accordance with the manufacturer’s instructions.

### TUNEL staining of retinal tissue

The detection of apoptotic cells in retinal tissue was carried out using the One Step TUNEL Assay Kit (KGA1408-20; KeyGEN). Frozen sections of eyes were then rinsed three times with 1× PBS and were subjected to TUNEL assay using according to the manufacturer’s protocol.

### Flow cytometry analysis

Cell apoptosis was assessed with the Annexin V-PE/7-AAD Apoptosis Detection Kit (A213-01; Vazyme). Cells were collected and mixed with 100 μl of 1×Binding Buffer to obtain a single-cell suspension. The solution was mixed with 5 μl of Annexin V-PE and 5 μl of 7-AAD Staining Solution, followed by incubation in the dark at room temperature for 10 min. After staining, the sample was analyzed by flow cytometry within 1 h.

### qRT-PCR

RNA extraction from retina or Müller cells was performed utilizing RNA isolater Total RNA Extraction Reagent (R401-01; Vazyme). The concentration of RNA was detected by NanoDrop One (Thermo, USA). The following primers were used: PTP1B forward 5′-CACAGTACGGCAGTTGGAGT-3′, reverse 5′-GCCAGGTGGTGTAGTGGAAA-3′; GAPDH forward 5′-GACAGCCGCATCTTCTTGTG-3′, reverse 5′-GGTAACCAGGCGTCCGATAC-3′; miR-125a-5p forward 5′-GCGTCCCTGAGAGACCCTTTAAC-3′, reverse 5′-AGTGCAGGGTCCGAGGTATT-3′; U6 forward 5′-CAAATCATGAGGCGTTCCAT-3′, reverse: 5′-AGTGCAGGGTCCGAGGTATT-3′. The expression of PTP1B mRNA was normalized to the level of GAPDH, while U6 was used as a reference gene for miR-125a-5p. The calculation was performed using the 2 − ΔΔCT method.

### Western blot

Cell lysates prepared using RIPA buffer were underwent SDS-PAGE gels and transferred to a 0.22 μm polyvinylidene difluoride blotting membrane. The membranes were incubated overnight at 4 °C with anti-CD81 (A4863; 1:1000; Abclonal), anti-TSG101 (A1692; 1:1000; Abclonal), anti-Calnexin (A4846; 1:1000; Abclonal), anti-occludin (A24601; 1:1000; Abclonal), anti-PTP1B (A23035; 1:2000; Abclonal), anti-BCL-2 (A11025; 1:1000; Abclonal), anti-BAX (A19684; 1:1000; Abclonal), anti-GAPDH (A19056; 1:50000; Abclonal), anti-GFAP (A19058; 1:1000; Abclonal), anti-LC3B (A19665; 1:1000; Abclonal), anti-P62 (A19700; 1:1000; Abclonal), anti-TOM20 (A19403; 1:2000; Abclonal), anti-PINK1 (A24745; 1:1000; Abclonal), anti-Parkin (A11172; 1:1000; Abclonal) antibodies, respectively. Following incubation with an HRP-conjugated secondary antibodies (AS014; 1:5000; Abclonal) at 37 °C for 1 h, ECL (RM02867; Abclonal) luminescence facilitated membrane imaging. The relative protein expression was assessed by calculating the gray ratio between the band representing the protein of target and the band representing GAPDH.

### Luciferase assay

The binding of miR-125a-5p to PTP1B was determined by a luciferase reporter gene assay. PTP1B 3ʹUTR sequence containing miR-125a-5p binding site and the corresponding mutant (MUT) sequence were cloned into pmirGLO dual-luciferase vector to obtain PTP1B-3ʹUTR wild type (WT) or MUT. The vectors were co-transfected with miR-125a-5p mimic or its NC into 293 T cells. At 2 days post-transfection, the luciferase activity was measured with dual luciferase reporter kits (RG027; Beyotime).

### Statistical analysis

All data were analyzed using GraphPad Prism 8 statistical software and expressed as mean ± SD. Comparisons between multiple groups were made using one-way ANOVA, and comparisons between two groups were made using Student ’s *t*-test. A difference was considered statistically significant if the value of *P* was less than 0.05. The experiment was conducted a minimum of three times.

## Supplementary information

Supplementary 1. miRNA sequencing data

Supplementary 2. Table S1 The hUCMSC-sEV-miRNAs with the minium free energy (MFE)

Supplementary 3. Full-length blots of Western blotting analysis

## Data Availability

The datasets analysed during the current study are available in the zenodo repository, 10.5281/zenodo.13959078. Further inquiries can be directed to the corresponding author.
