# Supplementary material for: MiR-125a-5p in MSC-derived small extracellular vesicles alleviates Müller cells injury in diabetic retinopathy by modulating mitophagy via PTP1B pathway
Source: Cell Death Discov. 2025 May 8;11:226. doi: 10.1038/s41420-025-02439-3 (PMC12062395; doi:10.1038/s41420-025-02439-3)
Supplement: Supplementary file 2 — Supplementary 2. Table S1 The hUCMSC-sEV-miRNAs with the minium free energy (MFE) [file 41420_2025_2439_MOESM2_ESM.docx]

**Table S1** The hUCMSC-sEV-miRNAs with the minium free energy (MFE), base paired with the 3'UTR of PTP1B mRNA. MFE was calculated by online software RNAhybrid (https://bibiserv.cebitec.uni-bielefeld.de/rnahybrid).

| miRNA Name | Sequence (5' to 3') | MFE (kcal/mol) |
| --- | --- | --- |
| miR-125b-5p | UCCCUGAGACCCUAACUUGUGA | -27.7 |
| miR-125a-5p | UCCCUGAGACCCUUUAACCUGUGA | -32.2 |
| miR-29c-3p | UAGCACCAUUUGAAAUCGGUUA | -23.2 |
| miR-29a-3p | UAGCACCAUCUGAAAUCGGUUA | -25.9 |
| miR-29b-3p | UAGCACCAUUUGAAAUCAGUGUU | -24.7 |
| miR-22-3p | AAGCUGCCAGUUGAAGAACUGU | -24.8 |

MFE: Minimum free energy
