# Supplementary material for: MiR-125a-5p in MSC-derived small extracellular vesicles alleviates Müller cells injury in diabetic retinopathy by modulating mitophagy via PTP1B pathway
Source: Cell Death Discov. 2025 May 8;11:226. doi: 10.1038/s41420-025-02439-3 (PMC12062395; doi:10.1038/s41420-025-02439-3)
Supplement: Supplementary file 3 — Supplementary 3. Full-length blots of Western blotting analysis [file 41420_2025_2439_MOESM3_ESM.pdf]

Uncutted Western Blot Gel

Fig. 1D

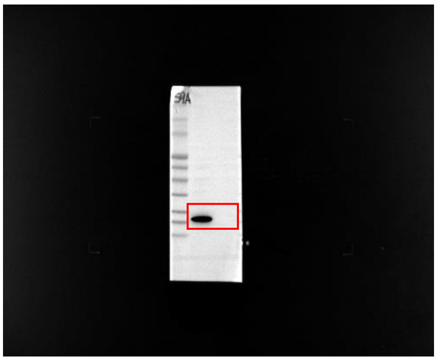

CD81

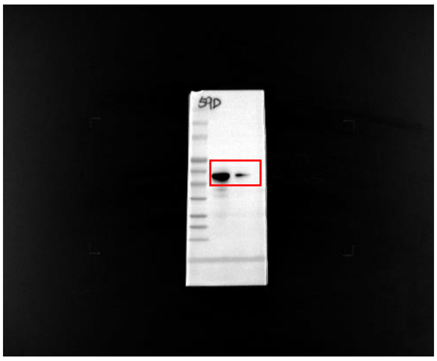

TSG101

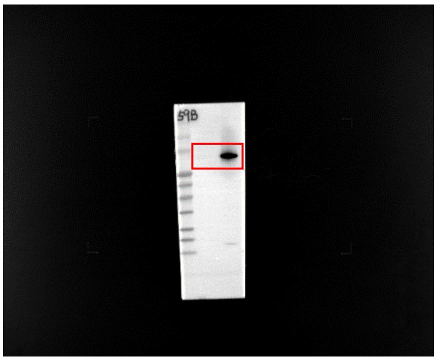

Calnexin

Fig. 1I

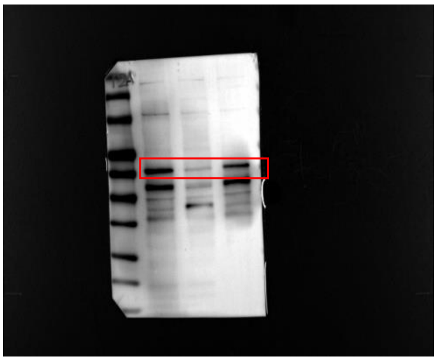

Occludin

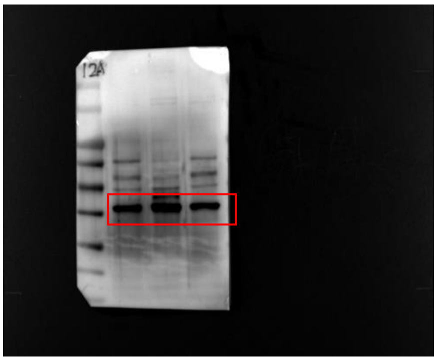

GADPH

Fig. 2E

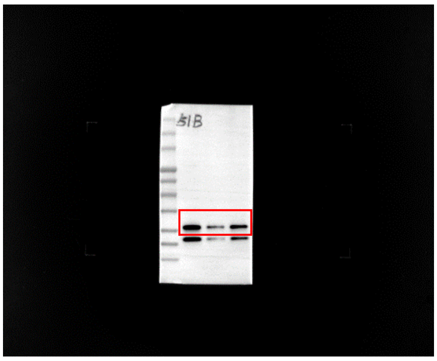

BCL-2

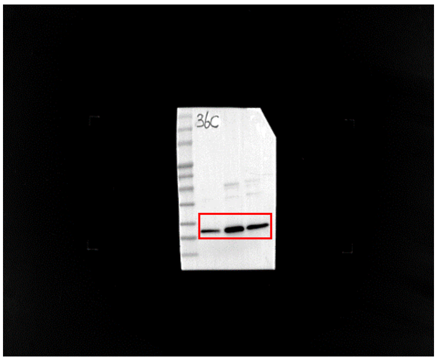

BAX

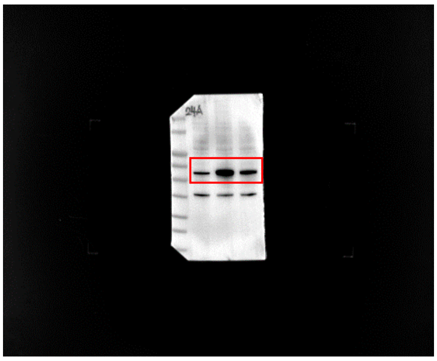

PTP1B

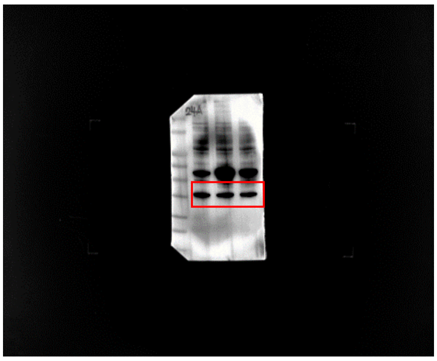

GAPDH

Fig. 3C

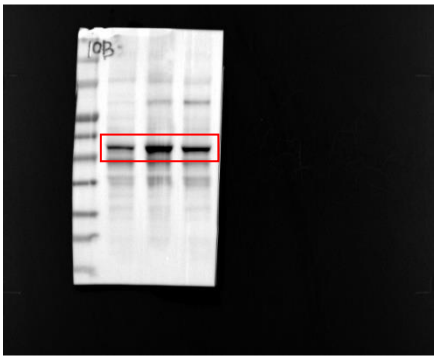

GFAP

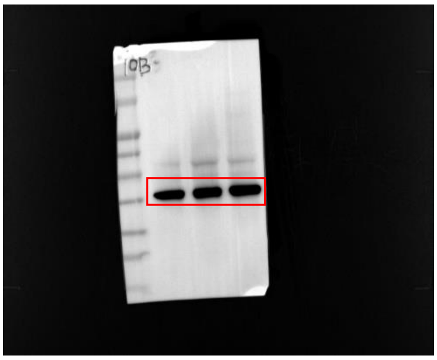

GAPDH

Fig. 4A

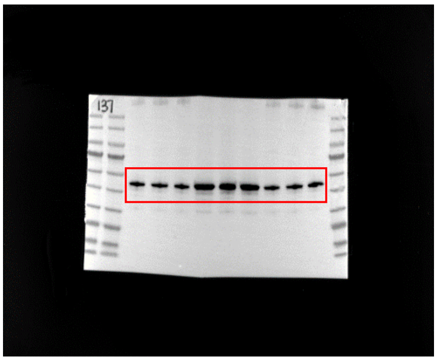

PTP1B

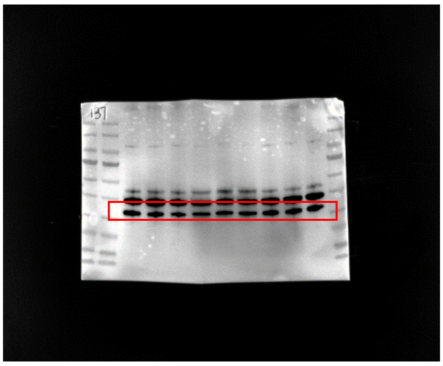

GAPDH

Fig. 4H

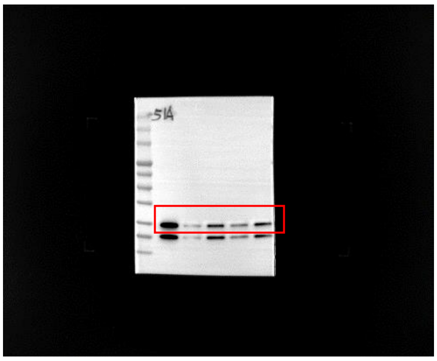

BCL-2

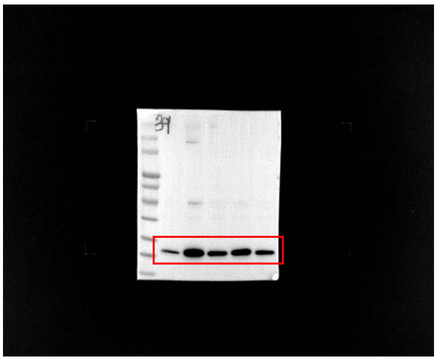

BAX

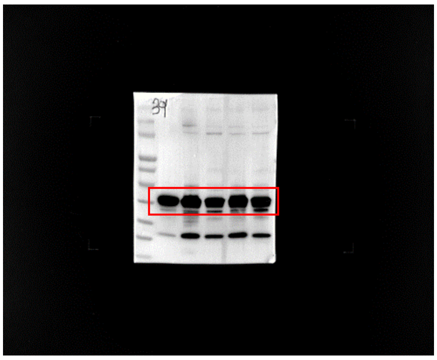

GAPDH

Fig. 6C

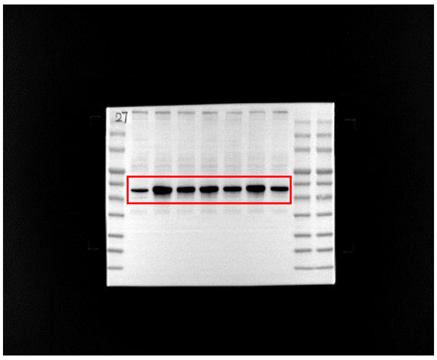

PTP1B

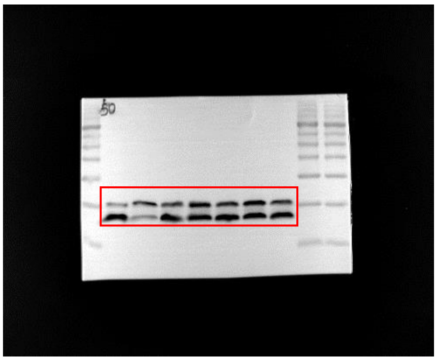

LC3BI/II

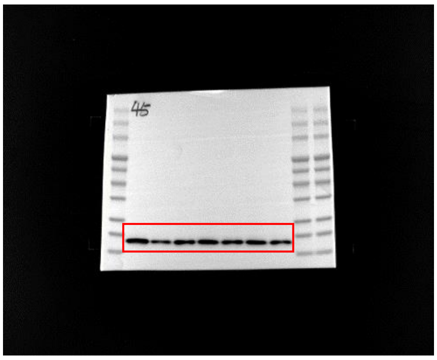

TOM20

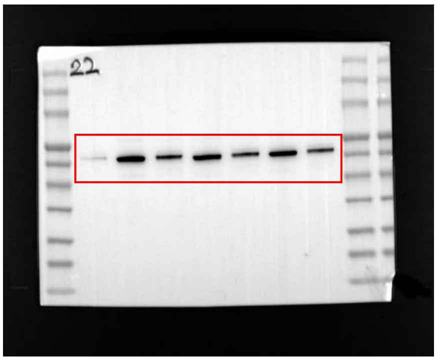

P62

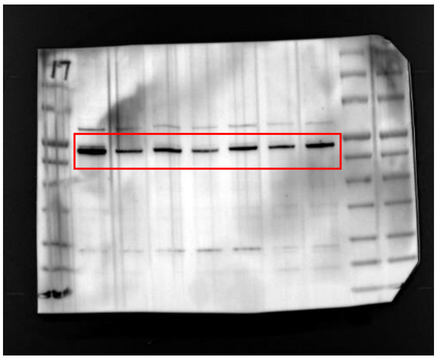

PINK1

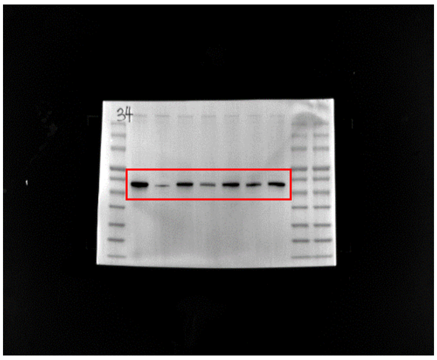

Parkin

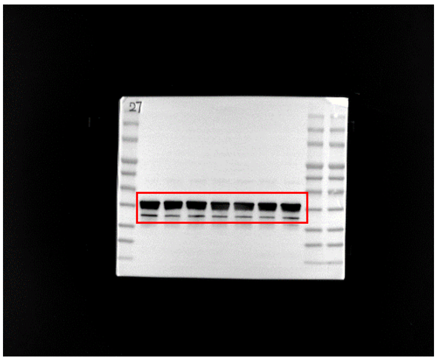

GAPDH
